# Risk factors and mortality of pulmonary embolism in COVID-19 patients: Evidence based on fifty observational studies

**DOI:** 10.1097/MD.0000000000029895

**Published:** 2022-11-11

**Authors:** Zhaoliang Fu, Gengshen Bai, Bingsheng Song, Yongbing Wang, Hui Song, Ming Ma, Junqiang Zhu, Zejun Zhang, Qinghong Kang

**Affiliations:** a Department of lnterventional, The Second People’s Hospital of Baiyin City, Baiyin, China; b Department of General Surgery, The Second People’s Hospital of Baiyin City, Baiyin, China; c Department of Respiratory Disease, The Second People’s Hospital of Baiyin City, Baiyin, China; d Department of Radiology, The Second People’s Hospital of Baiyin City, Baiyin, China.

**Keywords:** COVID-19, mortality, pulmonary embolism, risk factors

## Abstract

At present, many studies have described acute pulmonary embolism (PE) as a frequent and prognostically relevant complication of coronavirus disease 2019 (COVID-19) infection. Thus we performed the present analysis of 50 studies to evaluate the risk factors and mortality of PE in COVID-19 patients.

**Method:**

Databases including PubMed, Embase, Cochrane Library and Web of Science were searched to October, 2021. Odds ratio (OR), mean difference (MD) or standard MD was used to evaluate the outcomes. The primary outcomes were the difference of mortality between PE and non-PE COVID-19 patients as well as relevant risk factors of PE in COVID-19 patients. All statistical analyses were performed using the standard statistical procedures provided in Review Manager 5.2.

**Result:**

A total of 50 studies including 10053 patients were included in this meta-analysis. Our results indicated that COVID-19 patients with PE experienced significantly higher mortality than non-PE patients (21.9% vs. 10.7%), with a pooled OR of 2.21 (95% CI 1.30 – 3.76; *P* = .003). In addition, COVID-19 patients with PE also experienced more mechanical ventilation (MV) (OR 2.21; 95% CI 1.30 – 3.75; *P* = .003) and invasive mechanical ventilation (IMV) (OR 3.58; 95% CI 2.47 – 5.20; *P* < .0001) respectively. Univariate analysis (UVA) results indicated the Sequential Organ Failure Assessment (SOFA) score, time to deep venous thrombosis (DVT), nonintensive care unit (non-ICU) patients and no anticoagulation as risk factors of PE for COVID-19 patients. In addition, multivariate analysis also found that SOFA score, D-dimer, BMI > 30 kg/m^2^ and history of PE were risk factors of PE for COVID-19 patients.

**Conclusion:**

The present analysis indicated that PE increased the mortality of COVID-19 patients. Mechanical ventilation, especially invasive mechanical ventilation, is correlated with an increased incidence of PE in patients with COVID-19. The incidence of PE for COVID-19 patients may be multifactorial and further researches focused on risk factors were needed in the future.

## 1. Introduction

The outbreak of coronavirus disease 2019 (COVID-19) remains a severe public health emergency of international concern. Over the past months, several investigations have suggested an association between the COVID-19 pathogenesis and a pro-coagulant pattern that seems to be implicated in a higher risk of both arterial and venous thrombotic events.^[1–4]^ In this regard, acute pulmonary embolism (PE) has emerged as a potential severe complication of the infection and both American and European consensus statement have suggested general recommendations to deal with these clinical events.^[5,6]^

Many studies have reported a high incidence of PE in patients with COVID-19, ranging from 10.5% to 14.7% in patients who were admitted to general wards and from 23.4% to 24.7% in patients who were admitted to the intensive care unit (ICU).^[7–9]^ It was believed that complications of PE, such as pulmonary infection, pulmonary consolidation, pulmonary arterial hypertension and increasing of right heart load, increased or resulted in in-hospital death of COVID-19 patients.^[7]^ However, the influence of PE to mortality of COVID-19 patients was still unclear. In addition, the risk factors of PE are not evaluated at present in patients with COVID-19.

Thus we performed the present analysis to evaluate the risk factors and mortality of PE in COVID-19 patients. We aim to explore the risk factors of PE in COVID-19 patients and hope that this will be helpful to prophylaxis or diagnosis of PE in COVID-19 patients.

## 2. Methods

### 2.1. Search strategy and study selection

A systematic search of PubMed, Embase, Cochrane Library and Web of Science up to October, 2021 was conducted for relevant studies using a search strategy developed by a medical information specialist that involved controlled vocabulary and keywords related to our research question (e.g., “coronavirus disease”, “COVID-19”, “pulmonary embolism”, “PE”; “prognosis”, “outcome”, “survival”, “death”, “mortality”, “prevalence”, “risk factors”). The search strategy was limited to English language articles. Two assessors independently screened the titles and abstracts of each study. When a relevant study was identified, its full text was obtained for further evaluation. The full text of related references was also obtained for review.

### 2.2. Criteria for considering studies

We included studies if they met the following criteria: studies that: (1) compared the death or other outcomes between PE and non-PE patients with COVID-19; (2) explored the risk factors of PE in patients with COVID-19.

Studies were excluded if they met the following criteria: (1) experimental trial on animals or a nonhuman studies; (2) study population included non- COVID-19 patients; (3) study reported in the form of an abstract, letter, editorial, expert opinion, review, or case report; or (4) lack of sufficient data or failure to meet the inclusion criteria.

### 2.3. Quality assessment and data extraction

Two reviewers assessed the quality of each study using the 9-star Newcastle-Ottawa Scale (NOS).^[10]^ The scores were judged according to the three aspects of NOS of evaluation: selection, comparability, and outcome between the case group and control group. In addition, the risk of bias for each studies and the risk of bias across all studies were evaluated and shown with figures generated by RevMan 5.2 software.^[11]^

Baseline characteristics and outcomes from the included studies were extracted using a standardized extraction form. Key study characteristics including country, sample size, mean age, location, setting, end points and main outcomes were extracted. Data were extracted by 1 reviewer and then examined for accuracy and completeness by a second reviewer.

### 2.4. Data synthesis and statistical methods

The data of comparable outcomes between PE and non-PE patients with COVID-19 were combined-analyzed, using the standard statistical procedures provided in RevMan 5.2.^[11]^ Dichotomous data were measured with odds ratio (OR) and continuous variable data were measured with mean difference (MD). The heterogeneity between studies was evaluated by the chi-square-based Q statistical test,^[12]^ with *P*_*h*_ value and *I^2^* statistic, ranging from 0% to 100 %, to quantify the effect of heterogeneity. *P*_*h*_ ≤ 0.10 was deemed to represent significant heterogeneity,^[13]^ and pooled estimates were estimated using a random-effect model (the DerSimonian and Laird method^[14]^). On the contrary, if statistical study heterogeneity was not observed (*P*_*h*_ > 0.10), a fixed effects model (the Mantel–Haenszel method^[15]^) was used. The effects of outcome measures were considered to be statistically significant if pooled ORs with 95% CI did not overlap with 1 or pooled MDs with 95% CI did not overlap with 0.

This work has been reported in line with Preferred Reporting Items for Systematic Reviews and Meta-Analyses (PRISMA)^[16]^ and Assessing the methodological quality of systematic reviews (AMSTAR) Guidelines.^[17]^ Because this work in fact was a second analysis of previous study, the ethical approval for this study was not applicable.

## 3. Results

### 3.1. Included studies, study characteristics, and quality assessment

**Figure 1.**
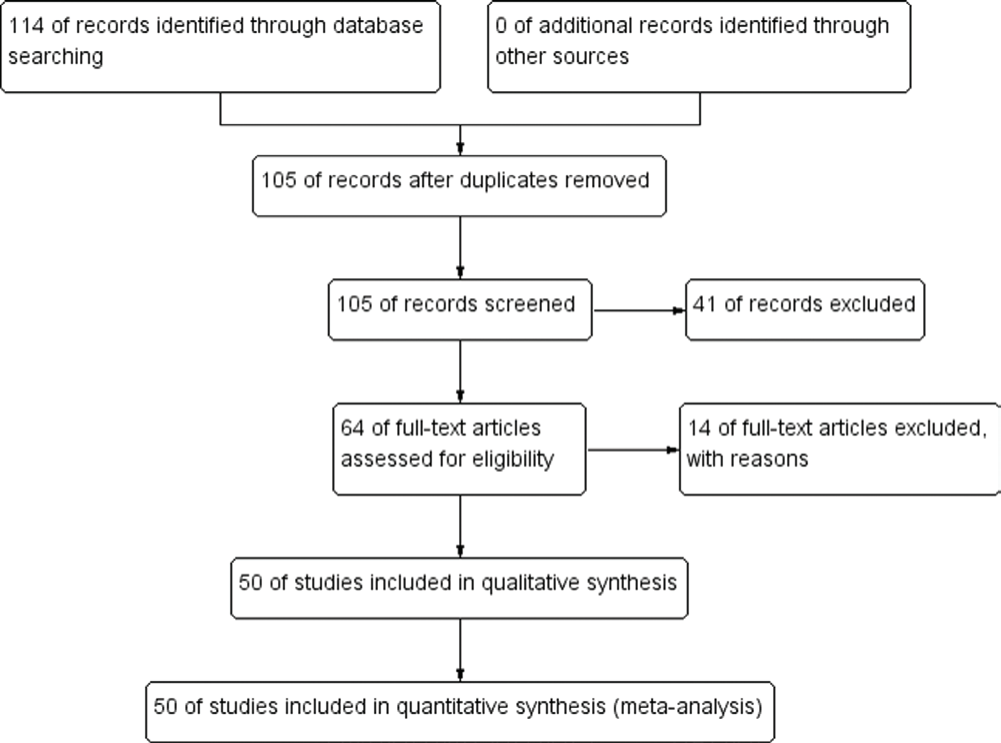
Flow diagram following the PRISMA template of the search strategy for the association between ALBI grade and the prognosis of patients with HCC.

At the beginning of the search, a total of 114 records of citations were obtained; 105 of records were reviewed further after duplicates were removed. By screening titles and abstracts, 41 studies were preliminarily excluded and the remaining 64 studies were retrieved in full text for further evaluation. After reading the full texts, 14 studies were excluded further. Eventually, 50 studies^[1,5,6,18–64]^ (10053 patients) were included in this systematic review and meta-analysis. Six studies were from USA, 14 from France, 8 from China, 3 from Switzerland, 5 from Spain. 15 studies were conducted by multicenter and the others by single center. The detailed search process and summary of studies are shown in the study flow diagram (Fig. 1). The other characteristics of each study are shown in Table 1.

**Table 1:** The characteristics of included studies in this meta-analysis.

Author/Year	Country	Sample size	Location	Setting	Age (mean ± SD, range)	Male (n)	Main outcomes
Alonso-Fernández A (2020)	Spain	30	Single-center	ICU, general wards	67.0 (63.0–73.0)	15	Incidence and risk factors of PE
Al-Samkari H (2020)	USA	400	Multicenter	ICU, general wards	60/ 65	228	Incidence and risk factors of PE
Artifoni M (2020)	France	71	Multicenter	General wards	64 (46.0–75)	43	Incidence and risk factors of PE
Bompard F (2020)	France	135	Multicenter	ICU	64 (64–76)	94	Incidence and risk factors of PE
Cattaneo M (2020)	Italy	64	Single center	General wards	70 (58–77.5)	35	Incidence and risk factors of PE
Chen J (2020)	China	25	Single center	General wards	65 (56.5–70)	15	Incidence and risk factors of PE
Chen S (2021)	China	88	Single center	ICU	63 (55–71)	54	Incidence and risk factors of DVT
Cui S (2020)	China	81	Single center	ICU	59.9 ± 14.1	37	Incidence and risk factors of PE
Demelo-Rodríguez P (2020)	Spain	156	Single center	General wards	68.1 ± 14.5	102	Incidence and risk factors of PE
Fang C (2020)	UK	93	Single center	ICU, general wards	62 (56–69)	60	Incidence and risk factors of PE
Fauvel C (2020)	France	1240	Multicenter	General wards	64 ± 17	559	Incidence of PE; death, admission to the ICU, invasive mechanical ventilation, and noninvasive ventilation
Fraissé M (2020)	France	92	Single center	ICU	61 (55–70)	73	Incidence and risk factors of PE
Freund Y (2020)	6 countries	3253	Multicenter	General wards	61.0 ± 19	1558	Association between PE and COVID-19
Galeano-Valle F (2020)	Spain	24	Single center	General wards	64.3 ± 14.4	14	Incidence and risk factors of PE
Gervaise A (2020)	France	72	Single center	General wards	62.3 ± 17.8	54	Incidence and risk factors of PE
Grandmaison G (2020)	Switzerland	29	Single center	ICU	64.6 ± 10.0	18	Incidence and risk factors of PE
Grillet F (2020)	France	100	Single center	ICU, General wards	66 ± 13	70	Incidence of PE and VTE
Hékimian G (2020)	France	51	Single center	ICU	51.9 ± 11.0	38	Incidence and risk factors of PE
Helms J (2020)	France	150	Multicenter	ICU	63 (53–71)	122	Incidence and risk factors of PE
Kerbikov O (2021)	Russia	75	Single center	ICU	63.4	36	Incidence and risk factors of PE
Klok FA (2020)	The Netherlands	184	Multicenter	ICU	64 ± 12	139	Incidence and risk factors of PE
Koleilat I (2021)	USA	135	Single center	General wards	63 ± 15	53	Potential risk factors for DVT
Le Jeune S (2021)	France	42	Single center	General wards	65 ± 19	55	Incidence of VTE
LeBrun DG (2020)	USA	59	Multicenter	ICU, General wards	68 (85–100)		Inpatient mortality; admission to the ICU, unexpected intubation, pneumonia, DVT, PE, MI, CVA, UTI, and transfusion
Léonard-Lorant I (2020)	France	106	Single center	ICU, General wards	62.5 ± 14.3	70	Incidence of PE
Llitjos JF (2020)	France	26	Multicenter	ICU	68 (51.7–74.5)	20	Incidence and risk factors of PE
Lodigiani C (2020)	Italy	362	Single center	ICU, General wards	66 (55–75)	264	Incidence and risk factors of PE
Longchamp A (2020)	Switzerland	25	Single center	ICU	68 ± 11	16	Incidence and risk factors of PE
Maatman TK (2020)	USA	109	Multicenter	ICU	61 ± 16	62	Incidence and risk factors of PE
Manjunath M (2020)	USA	23	Single center	ICU	61.7	15	Incidence and risk factors of PE
Marone EM (2020)	Italy	101	Single center	General wards	70 ± 10	58	Incidence and risk factors of PE
Menter T (2020)	Switzerland	21	Multicenter		76		Risk factors of mortality
Mestre-Gómez B (2021)	Spain	29	Single center	General wards	65 (56–73)	21	Cumulative incidence of PE; factors associated to the diagnosis of PE
Middeldorp S (2020)	The Netherlands	198	Single center	ICU, General wards	61 ± 14	130	Incidence and risk factors of PE
Mueller-Peltzer K (2020)	Germany	16	Single center	ICU	62 ± 8	13	Incidence and risk factors of PE
Poissy J (2020)	France	107	Single center	ICU	60.8 ± 14.0	78	Incidence and risk factors of PE
Poyiadji N (2020)	USA	328	Multicenter	ICU, General wards	59 ± 15	186	Incidence and risk factors of PE
Ren B (2020)	China	48	Multicenter	ICU	70 (62–80)	26	Incidence and risk factors of PE
Soumagne T (2020)	France Belgium	375	Multicenter	ICU	63.5 ± 10.1	288	Risk factors associated with PE
Thomas W (2020)	UK	63	Single center	ICU	59 ± 13	44	Thrombotic complications of patients
Trimaille A (2020)	France	289	Single center	General wards	62.2 ± 17.0	171	Incidence and risk factors of PE
Valle C (2021)	Italy	114	Multicenter	ICU, General wards	61 (51.2–66)	84	Incidence and risk factors of PE
van Dam LF (2020)	the Netherlands	23	Single center	General wards	63 ± 6.4	16	Incidence and risk factors of PE
van den Heuvel FMA (2020)	the Netherlands	51	Single center	General wards	63 (51–68)	41	the incidence of ventricular dysfunction and its relationship with biomarker
Ventura-Díaz S (2020)	Spain	242	Single center	General wards	66 ± 15	150	Incidence and risk factors of PE
Wang Y (2020)	China	237	Single center	General wards			Incidence and risk factors of PE
Whyte MB (2020)	UK	214	Single center	ICU, General wards	61.5 ± 2	129	Incidence and risk factors of PE
Wichmann D (2020)	Germany	12	Single center	General wards	73 (52–87)		Incidence of thromboembolic events
Yu Y (2020)	China	142	Single center	General wards	62 ± 12	110	Incidence and risk factors of PE
Zhang L (2020)	China	143	Single center	ICU	63 ± 14	74	Incidence and risk factors of PE

CVA= cerebrovascular accident, DVT= deep vein thrombosis, ICU= intensive care unit, MI = myocardial infarction, PE = pulmonary embolus, UTI = urinary tract infection, VTE = venous thrombus embolism.

**Figure 2.**
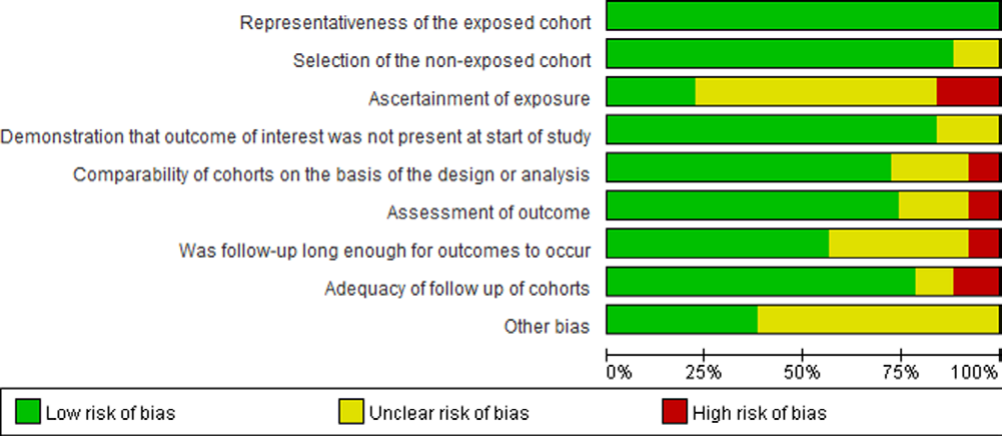
Risk of bias graph: review authors’ judgments about each risk of bias item presented as percentages across all included studies.

**Figure 3.**
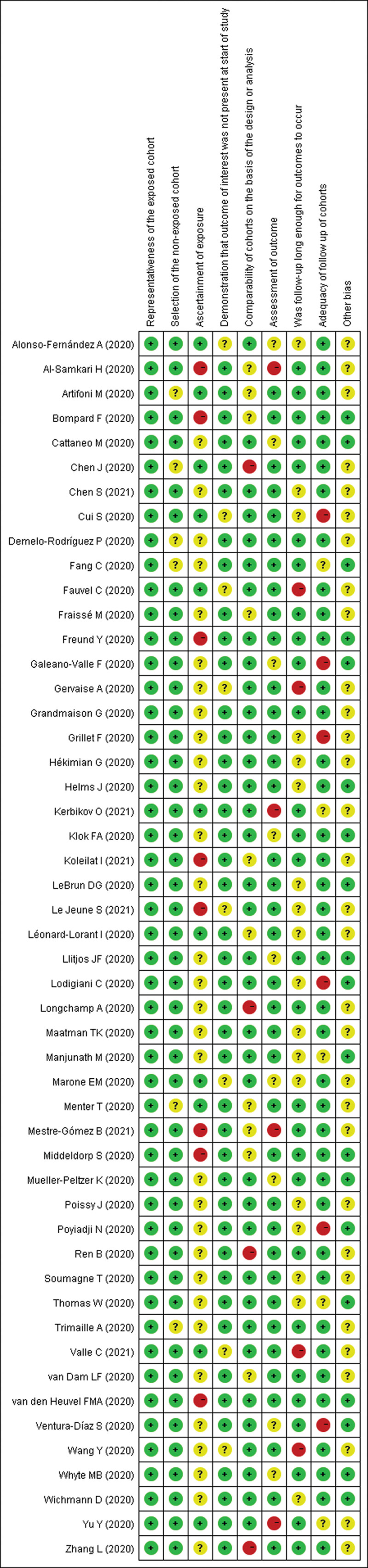
Risk of bias summary: review authors’ judgments about each risk of bias item for each included study.

Risk-of-bias graphs were generated to further identify the risk of bias of the including studies. The risk of bias for each study was presented as percentages across all included studies, and the risk-of-bias item for each included study was displayed (Fig. 2 and 3). The risk-of-bias graphs indicated generally low risk of selection and comparability. In addition, all studies experienced low risk of bias in “assessment of outcomes” item. A high risk of bias was mainly observed in “ascertainment of exposure” and “adequacy of follow-up of cohorts”. Unclear risk of bias was mainly observed in “ascertainment of exposure” and “other bias”.

### 3.2. The clinical outcomes of COVID-19 patients with PE

**Figure 4.**
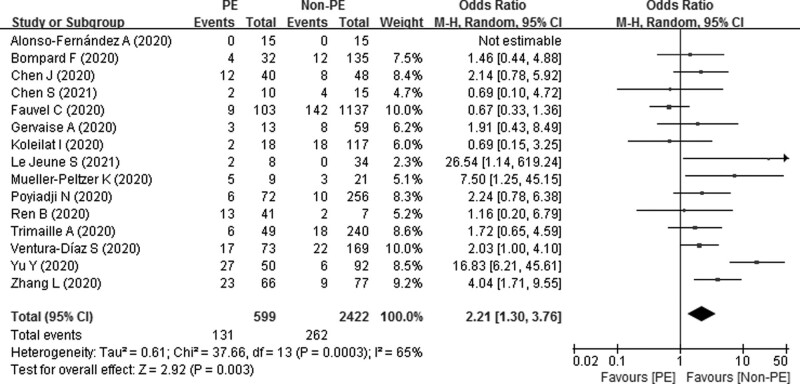
Forest plot of the mortality between PE and non-PE patients with COVID-19.

We compared the mortality of COVID-19 patients between PE and non-PE. As Figure 4 shows, our pooled results indicated that COVID-19 patients with PE experienced significantly higher mortality than non-PE patients (21.9% vs. 10.7%), with a pooled OR of 2.21 (95% CI 1.30 – 3.76; *P* = .003). As significant heterogeneity between studies was observed (*P* = .0003 and *I^2^* = 65%), the randomized effect model was used.

In addition, it was observed that COVID-19 patients with PE also experienced more ICU admission than non-PE, with a pooled OR of 2.79 (95% CI 1.88 – 4.13; *P* < .0001). However, no significant difference was found between PE and non-PE patients in the incidence of acute respiratory failure (OR 2.25; 95% CI 0.52 – 9.70; *P* = .28) and arrhythmia (OR 5.74; 95% CI 0.25 – 130.37; *P* = .27) (Table 2).

**Table 2:** The comparison of clinical outcomes in hospital between PE and non-PE patients with COVID-19.

Treatment	Sample size	Pooled results			Analytic effect model
		OR	95% CI	*P* value	
Acute respiratory failure	30	2.25	0.52, 9.70	0.28	Fixed effect model
Arrhythmia	30	5.74	0.25, 130.37	0.27	Fixed effect model
ICU admission	1405	2.79	1.88, 4.13	< 0.0001	Fixed effect model

COVID-19 = coronavirus disease 2019, ICU = intensive care unit, OR = odds ratio, PE = pulmonary embolism.

### 3.3. Anthropometric and clinical characteristics between PE and non-PE patients with COVID-19

In order to explore the difference of characteristics of COVID-19 patients with PE, we compared the characteristics between PE and non-PE patients with COVID-19. As Table 3 shows, compared with non-PE, COVID-19 patients with PE experienced longer time from illness onset to admission, with a pooled MD of 1.50 days (95% CI 0.45 – 2.55; *P* = .005). No significance was found in age (MD 3.99 years; 95% CI -0.77 – 8.76; *P* = .10), gender (OR 1.81; 95% CI 0.96 – 3.41; *P* = .07), BMI (MD -1.30 Kg/m^2^; 95% CI -3.42 – 0.82; *P* = .23), time to CTPA (MD 1.23 days; 95% CI -0.33 – 2.79; *P* = .12), hospitalization (MD 4.15 days; 95% CI -0.48 – 8.77; *P* = .08) respectively.

**Table 3:** The comparison of characteristics between PE and non-PE patients with COVID-19.

Characteristics	Sample size	Pooled results			Analytic effect model
		Estimate	95% CI	*P* value	
Age, yr	2206	MD 3.99	–0.77, 8.76	0.10	Random-effect model
Gender (men)	2095	OR 1.81	0.96, 3.41	0.07	Random-effect model
BMI, kg/m^2^	1519	MD –1.30	–3.42, 0.82	0.23	Random-effect model
Time to admission, day	1405	MD 1.50	0.45, 2.55	0.005	Fixed effect model
Time to CTPA, day	438	MD 1.23	–0.33, 2.79	0.12	Fixed effect model
Hospitalization, day	677	MD 4.15	–0.48, 8.77	0.08	Random-effect model

BMI = body mass index, COVID-19 = coronavirus disease 2019, CTPA = computed tomography pulmonary angiography, MD = mean difference, OR = odds ratio, PE = pulmonary embolism.

We found no difference between PE and non-PE COVID-19 patients in symptoms such as cough (OR 0.86; 95% CI 0.51 – 1.45; *P* = .57), fever (OR 1.71; 95% CI 0.88 – 3.33; *P* = .11), dyspnea (OR 1.27; 95% CI 0.73 – 2.22; *P* = .40), chest pain (OR 1.12; 95% CI 0.26 – 4.78; *P* = .88) (Table 4).

**Table 4:** The comparison of symptoms between PE and non-PE patients with COVID-19.

Symptoms	Sample size	Pooled results			Analytic effect model
		OR	95% CI	*P* value	
Cough	380	0.86	0.51, 1.45	0.57	Fixed effect model
Fever	380	1.71	0.88, 3.33	0.11	Fixed effect model
Dyspnea	245	1.27	0.73, 2.22	0.40	Fixed effect model
Chest pain	102	1.12	0.26, 4.78	0.88	Fixed effect model

PE = pulmonary embolism; OR = odds ratio; COVID-19 = coronavirus disease 2019.

The comparison of physical examination between PE and non-PE patients with COVID-19 similarly found no significant difference in respiratory rate (MD 2.00 breaths/min; 95% CI -4.69 – 8.69; *P* = .56), heart rate (MD -2.00 beats/min; 95% CI -17.54 – 13.54; *P* = .80), systolic BP (MD -2.00 mm Hg; 95% CI -17.56 – 13.56; *P* = .80), diastolic BP (MD 4.00 mm Hg; 95% CI -10.88 – 18.88; *P* = .60), temperature (MD 0.00 ˚C; 95% CI -1.57 – 1.57; *P* = 1.0), and lower limb edema (OR 0.56; 95% CI 0.02 – 17.92; *P* = .74) (Table 5).

**Table 5:** The comparison of physical examination between PE and non-PE patients with COVID-19.

Examination	Sample size	Pooled results			Analytic effect model
		Estimate	95% CI	*P* value	
Respiratory rate (breaths/min)	30	MD 2.00	–4.69, 8.69	0.56	Fixed effect model
Heart rate (beats/min)	30	MD -2.00	–17.54, 13.54	0.80	Fixed effect model
Systolic BP (mm Hg)	30	MD -2.00	–17.56, 13.56	0.80	Fixed effect model
Diastolic BP (mm Hg)	30	MD 4.00	–10.88, 18.88	0.60	Fixed effect model
Temperature, ˚C	30	MD 0.00	–1.57, 1.57	1.00	Fixed effect model
Lower limb edema	16	OR 0.56	0.02, 17.92	0.74	Fixed effect model

BP = blood pressure, COVID-19 = coronavirus disease 2019, OR = odds ratio, PE = pulmonary embolism.

We compared relevant risk factors between PE and non-PE patients with COVID-19. As Table 6 shows, no significant difference between PE and non-PE patients with COVID-19 was found in diabetes mellitus (OR 0.98; 95% CI 0.66 – 1.45), cardiovascular disease (OR 1.18; 95% CI 0.69 – 2.01), chronic respiratory disease (OR 1.28; 95% CI 0.67 – 2.43), varicose veins (OR 3.21; 95% CI 0.12 – 85.20), chronic venous insufficiency (OR 0.31; 95% CI 0.01 – 8.28), neoplasm (OR 3.21; 95% CI 0.12 – 85.20), previous VTE, chronic heart failure, ischemic heart disease, pregnancy or puerperium, obesity (OR 0.73; 95% CI 0.15 – 3.49), 1 or more known risk factors for PE (OR 1.51; 95% CI 0.11 – 21.35).

**Table 6:** The comparison of relevant PE risk factors between PE and non-PE patients with COVID-19.

Relevant factors	Sample size	Pooled results			Analytic effect model
		OR	95% CI	*P* value	
Diabetes mellitus	1560	0.98	0.66, 1.45	0.90	Fixed effect model
Cardiovascular disease	1425	1.18	0.69, 2.01	0.54	Fixed effect model
Chronic respiratory disease	1435	1.28	0.67, 2.43	0.45	Fixed effect model
Varicose veins	30	3.21	0.12, 85.20	0.49	Fixed effect model
Chronic venous insufficiency	30	0.31	0.01, 8.28	0.49	Fixed effect model
Neoplasm	30	3.21	0.12, 85.20	0.49	Fixed effect model
Previous VTE	190	0.92	0.22, 3.92	0.91	Fixed effect model
Chronic heart failure	30	3.21	0.12, 85.20	0.49	Fixed effect model
Ischemic heart disease	30	0.46	0.04, 5.75	0.55	Fixed effect model
Pregnancy or puerperium	30	0.31	0.01, 8.28	0.49	Fixed effect model
Obesity	30	0.73	0.15, 3.49	0.69	Fixed effect model
One or more known risk factors for PE	1383	1.51	0.11, 21.35	0.76	Random-effect model

COVID-19 = coronavirus disease 2019, OR = odds ratio, PE = pulmonary embolism, VTE = venous thrombus embolism.

We also compared the incidence of PE in patients with COVID-19 of different treatment in hospital. The results indicated no significant difference between PE and non-PE patients with COVID-19 in azithromycin (OR 0.22; 95% CI 0.04 – 1.11), hydroxychloroquine (OR 2.15; 95% CI 0.17 – 26.67), lopinavir + ritonavir (OR 0.76; 95% CI 0.18 – 3.24), tocilizumab (OR 0.42; 95% CI 0.09 – 1.92), other biological therapy (OR 3.21; 95% CI 0.12 – 85.20) and systemic corticosteroids (OR 1.03; 95% CI 0.33 – 3.25) (Table 7).

**Table 7:** The comparison of treatment in hospital between PE and non-PE patients with COVID-19.

Treatment	Sample size	Pooled results			Analytic effect model
		OR	95% CI	*P* value	
Azithromycin	30	0.22	0.04, 1.11	0.07	Fixed effect model
Hydroxychloroquine	30	2.15	0.17, 26.67	0.55	Fixed effect model
Lopinavir + Ritonavir	30	0.76	0.18, 3.24	0.71	Fixed effect model
Tocilizumab	30	0.42	0.09, 1.92	0.26	Fixed effect model
Other biological therapy	30	3.21	0.12, 85.20	0.49	Fixed effect model
Systemic corticosteroids	55	1.03	0.33, 3.25	0.95	Fixed effect model

COVID-19 = coronavirus disease 2019, OR = odds ratio, PE = pulmonary embolism.

### 3.4. Comparison of oxygen therapy in hospital between PE and non-PE patients with COVID-19

**Figure 5.**
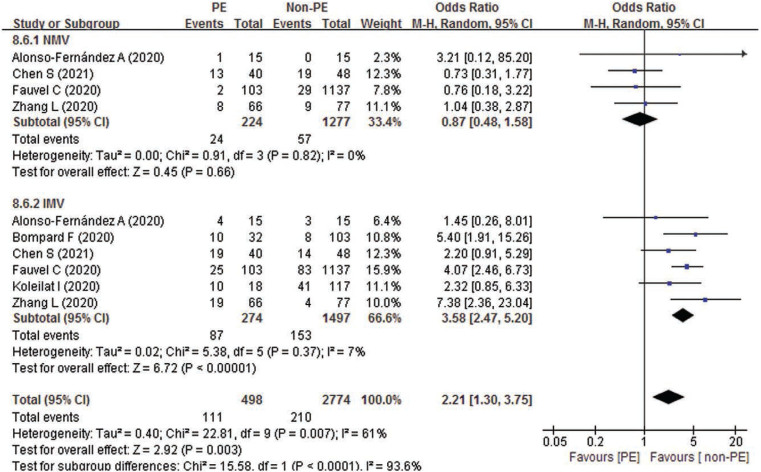
Forest plot of the mechanical ventilation use between PE and non-PE patients with COVID-19.

In order to explore the risk factors of PE in COVID-19 patients, we also compared the oxygen therapy in hospital between PE and non-PE patients with COVID-19. Our pooled results indicated that COVID-19 patients with PE experienced more mechanical ventilation (MV) (OR 2.21; 95% CI 1.30 – 3.75; *P* = .003) and invasive mechanical ventilation (IMV) (OR 3.58; 95% CI 2.47 – 5.20; *P* < .0001) respectively (Fig. 5). However, no significant difference was observed in maximum FiO2 (MD -0.27; 95% CI -0.89 – 0.34; *P* = .39), high-flow nasal cannula (HFNC) (OR 0.50; 95% CI 0.20 – 1.23; *P* = .13) and noninvasive mechanical ventilation (NMV) (OR 0.87; 95% CI 0.48 – 1.58; *P* = .66) (Table 8).

**Table 8:** The comparison of oxygen therapy in hospital between PE and non-PE patients with COVID-19.

Treatment	Sample size	Pooled results			Analytic effect model
		Estimate	95% CI	*P* value	
Maximum FiO_2_	1270	MD –0.27	–0.89, 0.34	0.39	Random-effect model
HFNC	118	OR 0.50	0.20, 1.23	0.13	Fixed effect model
NMV	1501	OR 0.87	0.48, 1.58	0.66	Random-effect model
IMV	1771	OR 3.58	2.47, 5.20	<0.0001	Random-effect model
MV	3272	OR 2.21	1.30, 3.75	0.003	Random-effect model

COVID-19 = coronavirus disease 2019, HFNC = high-flow nasal cannula, IMV = invasive mechanical ventilation, MD = mean difference, MV = Mechanical ventilation, NMV = noninvasive mechanical ventilation, OR = odds ratio, PE = pulmonary embolism.

### 3.5. Laboratory findings between PE and non-PE patients with COVID-19

We compared the laboratory indicators between PE and non-PE COVID-19 patients. Our pooled analysis indicated that compared to non-PE, COVID-19 patients with PE had higher baseline and peak serum D-dimer, with pooled MDs of 5.98 μg/mL (95% CI 4.15 – 7.81; *P* < .0001) and 1.10 μg/mL (95% CI 0.13 – 2.07; *P* = .03) respectively. In addition, COVID-19 patients with PE had higher NT-pro BNP (MD 94.24 pg/mL; 95% CI 45.21 – 143.27; *P* = .0002), hs Troponin I (MD 5.00 ng/L; 95% CI 1.01 – 8.99; *P* = .01), but lower albumin (MD -3.58 g/L; 95% CI -5.18 to -1.98; *P* < .0001) (Table 9).

**Table 9:** The comparison of laboratory findings between PE and non-PE patients with COVID-19.

Laboratory indicators	Sample size	Pooled results			Analytic effect model
		Estimate	95% CI	*P* value	
Serum D-dimer, μg/mL					
Baseline	2703	MD 5.98	4.15, 7.81	< 0.0001	Random-effect model
Peak	30	MD 1.10	0.13, 2.07	0.03	Fixed effect model
Prior to CTPA	30	MD 1.00	–0.17, 2.17	0.09	Fixed effect model
Ferritin, ng/mL					
Baseline	165	MD -213.68	–916.29, 488.93	0.98	Random-effect model
Peak	30	MD -20.82	–1373.57, 1331.94	0.07	Fixed effect model
Prior to CTPA	30	MD -1150.0	–2390.38, 90.38	0.71	Fixed effect model
Platelets, 10^3^/μL					
Baseline	1616	MD 0.49	–39.76, 40.75	0.98	Random-effect model
Peak	30	MD -55.0	–147.31, 37.31	0.24	Fixed effect model
Prior to CTPA	30	MD 12.0	–24.84, 48.84	0.52	Fixed effect model
Lymphocytes					
Baseline	1641	SMD -0.12	-0.37, 0.14	0.37	Random-effect model
Peak	30	SMD -0.34	–1.06, 0.38	0.35	Fixed effect model
Prior to CTPA	30	SMD -0.68	–1.42, 0.06	0.07	Fixed effect model
NLR					
Baseline	165	MD 2.17	–0.54, 4.89	0.12	Fixed effect model
Peak	30	MD 3.70	–5.15, 12.55	0.41	Fixed effect model
Prior to CTPA	30	MD 2.20	–0.25, 4.65	0.08	Fixed effect model
IL-6, pg/mL	207	MD -2.23	–33.02, 28.56	0.89	Fixed effect model
NT-pro BNP, pg/mL	1383	MD 94.24	45.21, 143.27	0.0002	Fixed effect model
hs Troponin I, ng/L	30	MD 5.00	1.01, 8.99	0.01	Fixed effect model
Fibrinogen, mg/dL	1447	MD 1.96	–1.95, 5.87	0.33	Fixed effect model
Albumin, g/L	88	MD -3.58	–5.18, –1.98	<0.0001	Fixed effect model
SOFA score	135	MD -1.00	–4.03, 2.03	0.52	Fixed effect model

COVID-19 = coronavirus disease 2019, CTPA = computed tomography pulmonary angiography, MD = mean difference, NLR = neutrophil to lymphocyte ratio, PE = pulmonary embolism, SMD = standardized mean difference, SOFA = Sequential Organ Failure Assessment.

However, no significant difference was found in ferritin, platelets, lymphocytes, NLR, IL-6 (MD -2.23 pg/mL; 95% CI -33.02 – 28.56; *P* = .89), fibrinogen (MD 1.96 mg/dL; 95% CI -1.95 – 5.87; *P* = .33) and SOFA score (MD -1.00; 95% CI -4.03 – 2.03; *P* = .52) (Table 9).

### 3.6. Risk factors associated with PE for patients with COVID-19

Univariate analysis (UVA) results indicated SOFA score (OR 1.87; 95% CI 1.39 – 2.52; *P* < .0001), time to DVT (OR 1.04; 95% CI 1.01 – 1.07; *P* = .009), non-ICU patients (OR 6.50; 95% CI 2.10 – 20.12; *P* = .001), no anticoagulation (OR 3.00; 95% CI 1.10 – 8.18; *P* = .03) and dyslipidemias (OR 9.06; 95% CI 1.88 – 43.67; *P* = .006) as risk factors of PE for COVID-19 patients. In addition, multivariate analysis (MVA) also found that SOFA score (OR 2.07; 95% CI 1.38 – 3.11; *P* = .0004), D-dimer (OR 2.82; 95% CI 1.05 – 7.58; *P* = .04), BMI > 30 kg/m^2^ (OR 2.70; 95% CI 1.30 – 5.61; *P* = .008) and history of PE (OR 3.50; 95% CI 1.20 – 10.21; *P* = .02) were risk factors of PE for COVID-19 patients. Inversely, MVA indicated that COVID-19 patients receiving statin therapy had negative correlation to PE, with a pooled OR of 0.40 (95% CI 0.20 – 0.80; *P* = .01). However, age, gender, PaO2/FiO2 ratio, and hypertension were not indicated as risk factors of PE for patients with COVID-19 (Table 10).

**Table 10:** The pooled results of univariate and multivariate analysis of risk factors associated with PE for patients with COVID-19.

Risk factors	Pooled UVA results			Pooled MVA results		
	OR	95% CI	*P* value	OR	95% CI	*P* value
Age	1.01	0.86, 1.19	0.90	1.00	0.81, 1.23	1.0
Gender (male)	1.09	0.46, 2.58	0.84			
SOFA score	1.87	1.39, 2.52	<0.0001	2.07	1.38, 3.11	0.0004
D-dimer	3.34	0.39, 28.82	0.27	2.82	1.05, 7.58	0.04
PaO_2_/FiO_2_ ratio (> 150)	0.54	0.23, 1.27	0.16			
Time to DVT	1.04	1.01, 1.07	0.009	1.04	1.00, 1.08	0.05
Non-ICU patients	6.50	2.10, 20.12	0.001			
No Anticoagulation	3.00	1.10, 8.18	0.03			
Dyslipidemia	9.06	1.88, 43.67	0.006			
BMI > 30 kg/m^2^				2.70	1.30, 5.61	0.008
Statin therapy				0.40	0.20, 0.80	0.01
History of PE				3.50	1.20, 10.21	0.02
Hypertension				0.50	0.20, 1.25	0.14

BMI = body mass index, COVID-19 = coronavirus disease 2019, DVT = deep vein thrombosis, MVA = multivariate analysis, OR = odds ratio, PE = pulmonary embolism, SOFA = sequential organ failure assessment, UVA = univariate analysis.

### 3.7. Publication bias

Funnel plots were conducted for assessing the publication bias of included literatures and we could roughly assess the publication bias by seeing whether their shapes were of any obvious asymmetry. The funnel plots showed no clear evidence of publication bias for mortality between PE and non-PE patients with COVID-19 (see supplemental digital content, http://links.lww.com/MD/G909).

## 4. Discussion

PE is a life-threatening complication in patients with COVID-19, and given the data presented by previous studies, patients with COVID-19 always experienced a high incidence of PE and mortality.^[7,8]^ However, the risk factors of PE for patients with COVID-19 are still unclear. Thus, we conducted the present analysis with 50 observational studies including 10053 patients in order to explore the relevant risk factors of PE for patients with COVID-19.

Our results indicated that COVID-19 patients with PE always experienced more ICU admission, longer time from illness onset to admission, more mechanical ventilation and IMV, higher baseline and peak serum D-dimer, higher NT-pro BNP and hs Troponin I, but lower albumin. In addition, SOFA score, time to DVT, non-ICU patients, no anticoagulation and dyslipidemias was indicated as risk factors of PE for COVID-19 patients. Multivariate analysis also found that SOFA score, D-dimer, BMI > 30 kg/m^2^ and history of PE may be independent risk factors of PE for COVID-19 patients. COVID-19 patients receiving statin therapy may reduce the risk of PE.

To the best of our knowledge, the present analysis is the first systematic review and meta-analysis designed to focus on the clinical relevant risk factors instead of the prevalence of PE in patients with COVID-19.^[7–9,65,66]^ However, prior to our analysis, 1 meta-analysis was performed to summarize evidence on the incidence of clinically relevant VTE—defined as VTE excluding isolated subsegmental PE and distal deep vein thrombosis—in adult critically ill patients with COVID-19.^[67]^ The author reported longer mean ICU stay, advanced age and overweight, critical illness, immobility were associated with increased VTE risk.^[67]^ This was in line with our results to a large extent.

Severe COVID-19 disease is accompanied by excessive cytokine release, which in turn activates the coagulation cascade, resulting in typical laboratory alterations such as elevated fibrinogen and D-dimer levels.^[68]^ Thus we compared the laboratory indicators between PE and non-PE patients aiming to found the difference and possible risk factors of PE in COVID-19 patients. As a results, serum D-dimer, higher NT-pro BNP and hs Troponin I as well as albumin level were found significant difference and may be associated with the incidence of PE.

Several relevant limitations of our work need to be recognized. First, as our results indicated that statin therapy may beneficial to PE and no anticoagulation may increase the incidence of PE for COVID-19 patients, some patients included in our included studies may receive this treatment. However, these studies did not report this part of patients, which resulted in our failure to perform subgroup analysis further. To date, only 1 prospective, randomized, controlled trial has compared different anticoagulation regimens in critically ill patients (n = 20) with COVID-19.^[69]^ Therefore, it seems unlikely that a meta-analysis could shed light on this important question at this point. In line with this, a recently published Cochrane review concluded that there is currently insufficient evidence to determine the risks and benefits of anticoagulation in patients with COVID-19.^[70]^ Second, we observed substantial heterogeneity among studies that—apart from distinct outcome definitions—may have been caused by differences in study designs and settings. In particular, the absence of uniform diagnostic procedures to detect PE needs to be borne in mind when interpreting the results of our study. Furthermore, we cannot exclude that the different included patient cohorts and different treatment strategies used in studies might have resulted in distinct PE risks. Third, the inherent limitations of retrospective data reporting applied to the majority of the included studies. This is a likely explanation for our finding that all of the included studies had a moderate to high risk of bias.

## 5. Conclusion

In conclusion, the present study summarizes the globally available risk factors of PE in patients with COVID-19. We calculated and compared the mortality of COVID-19 patients and found more than twofold mortality in PE than non-PE patients. Though it is multifactorial, we found several relevant risk factors of PE in patients with COVID-19 which may be helpful to clinical precaution and patients with these risk factors should be vigilant for PE.

## Author contributions

The authors on this paper all participated in study design. All authors read, critiqued and approved the manuscript revisions as well as the final version of the manuscript. Also, all authors participated in a session to discuss the results and consider strategies for analysis and interpretation of the data before the final data analysis was performed and the manuscript written. All authors have the appropriate permissions and rights to the reported data.

Ethical approval: Not applicable.

## Supplementary Material



## References

[1] Lodigiani C. Venous and arterial thromboembolic complications in COVID-19 patients admitted to an academic hospital in Milan, Italy. Thromb Res. 2020;191:9–14. 32353746 10.1016/j.thromres.2020.04.024 PMC7177070

[2] Silva BV. Pulmonary embolism and COVID-19: a comparative analysis of different diagnostic models performance. Am J Emerg Med. 2021;50:526–31. 34547695 10.1016/j.ajem.2021.09.004 PMC8423667

[3] van Twist DJL Luu IHY. Pulmonary embolism in COVID-19: the actual prevalence remains unclear. Radiol. 2021;299:E254. 10.1148/radiol.2021204671 PMC7971425 33724068

[4] Woodard PK. Pulmonary thromboembolism in COVID-19. Radiology. 2021;298:E107–8. 33325809 10.1148/radiol.2020204175 PMC7745995

[5] Klok FA. Confirmation of the high cumulative incidence of thrombotic complications in critically ill ICU patients with COVID-19: AN updated analysis. Thromb Res. 2020;191:148–50. 32381264 10.1016/j.thromres.2020.04.041 PMC7192101

[6] Léonard-Lorant I Delabranche X. Acute pulmonary embolism in patients with COVID-19 at CT angiography and relationship to d-Dimer levels. Radiol. 2020;296:E189–e191. 10.1148/radiol.2020201561 PMC7233397 32324102

[7] Liao SC. Incidence and mortality of pulmonary embolism in COVID-19: a systematic review and meta-analysis. Crit Care. 2020;24:464. 32718343 10.1186/s13054-020-03175-z PMC7384281

[8] Roncon L. Incidence of acute pulmonary embolism in COVID-19 patients: systematic review and meta-analysis. Eur J Intern Med. 2020;82:29–37. 32958372 10.1016/j.ejim.2020.09.006 PMC7498252

[9] Suh YJ Hong H. Pulmonary embolism and deep vein thrombosis in COVID-19: a systematic review and meta-analysis. Radiol. 2021;298:E70–e80 10.1148/radiol.2020203557 PMC7745997 33320063

[10] Stang A. Critical evaluation of the Newcastle-Ottawa scale for the assessment of the quality of nonrandomized studies in meta-analyses. Eur J Epidemiol. 2010;25:603–5. 20652370 10.1007/s10654-010-9491-z

[11] Review Manager (RevMan) [Computer Program]. Version 5.2. Copenhagen: The Nordic Cochrane Centre, The Cochrane Collaboration, 2012.

[12] Lau J Ioannidis JP Schmid CH. Quantitative synthesis in systematic reviews. Ann Intern Med. 1997;127:820–6. 9382404 10.7326/0003-4819-127-9-199711010-00008

[13] University of York Centre for Reviews and Dissemination. Systematic Reviews: CRD’s Guidance for Undertaking Reviews in Health Care. York: CRD, University of York, 2009.

[14] DerSimonian R Laird N. Meta-analysis in clinical trials revisited. Contemp Clin Trials. 2015;45(Pt A):139–45. 26343745 10.1016/j.cct.2015.09.002 PMC4639420

[15] Mantel N Haenszel W. Statistical aspects of the analysis of data from retrospective studies of disease. J Natl Cancer Inst. 1959;22:719–48. 13655060

[16] Moher D. Preferred reporting items for systematic reviews and meta-analyses: the PRISMA statement. BMJ. 2009;339:b2535. 19622551 10.1136/bmj.b2535 PMC2714657

[17] Shea BJ. AMSTAR 2: a critical appraisal tool for systematic reviews that include randomised or non-randomised studies of healthcare interventions, or both. BMJ. 2017;358:j4008. 28935701 10.1136/bmj.j4008 PMC5833365

[18] Alonso-Fernández A. Prevalence of pulmonary embolism in patients with COVID-19 pneumonia and high D-dimer values: a prospective study. PLoS One. 2020;15:e0238216e0238216. 32841275 10.1371/journal.pone.0238216 PMC7447036

[19] Al-Samkari H. COVID-19 and coagulation: bleeding and thrombotic manifestations of SARS-CoV-2 infection. Blood. 2020;136:489–500. 32492712 10.1182/blood.2020006520 PMC7378457

[20] Artifoni M. Systematic assessment of venous thromboembolism in COVID-19 patients receiving thromboprophylaxis: incidence and role of D-dimer as predictive factors. J Thromb Thrombolysis. 2020;50:211–6. 32451823 10.1007/s11239-020-02146-z PMC7246965

[21] Bompard F. Pulmonary embolism in patients with COVID-19 pneumonia. Eur Respir J. 2020;56:2001365. 32398297 10.1183/13993003.01365-2020 PMC7236820

[22] Cattaneo M. Pulmonary embolism or pulmonary thrombosis in COVID-19? is the recommendation to use high-dose heparin for thromboprophylaxis justified? Thromb Haemost. 2020;120:1230–2. 32349132 10.1055/s-0040-1712097 PMC7516356

[23] Chen J. Characteristics of acute pulmonary embolism in patients with COVID-19 associated pneumonia from the City of Wuhan. Clin Appl Thromb Hemost. 2020;26:1076029620936771076029620936772. 10.1177/1076029620936772 PMC7391435 32726134

[24] Chen S. DVT incidence and risk factors in critically ill patients with COVID-19. J Thromb Thrombolysis. 2021;51:33–9. 32607652 10.1007/s11239-020-02181-w PMC7324310

[25] Cui S. Prevalence of venous thromboembolism in patients with severe novel coronavirus pneumonia. J Thromb Haemost. 2020;18:1421–4. 32271988 10.1111/jth.14830 PMC7262324

[26] Demelo-Rodríguez P. Incidence of asymptomatic deep vein thrombosis in patients with COVID-19 pneumonia and elevated D-dimer levels. Thromb Res. 2020;192:23–6. 32405101 10.1016/j.thromres.2020.05.018 PMC7219400

[27] Fang C. Extent of pulmonary thromboembolic disease in patients with COVID-19 on CT: relationship with pulmonary parenchymal disease. Clin Radiol. 2020;75:780–8. 32684301 10.1016/j.crad.2020.07.002 PMC7351373

[28] Fauvel C. Pulmonary embolism in COVID-19 patients: a French multicentre cohort study. Eur Heart J. 2020;41:3058–68. 32656565 10.1093/eurheartj/ehaa500 PMC7528952

[29] Fraissé M. Thrombotic and hemorrhagic events in critically ill COVID-19 patients: a French monocenter retrospective study. Crit Care. 2020;24:275. 32487122 10.1186/s13054-020-03025-y PMC7265664

[30] Freund Y. Association between pulmonary embolism and COVID-19 in emergency department patients undergoing computed tomography pulmonary angiogram: the PEPCOV international retrospective study. Acad Emerg Med. 2020;27:811–20. 32734624 10.1111/acem.14096

[31] Galeano-Valle F. Antiphospholipid antibodies are not elevated in patients with severe COVID-19 pneumonia and venous thromboembolism. Thromb Res. 2020;192:113–5. 32425261 10.1016/j.thromres.2020.05.017 PMC7227496

[32] Gervaise A. Acute pulmonary embolism in non-hospitalized COVID-19 patients referred to CTPA by emergency department. Eur Radiol. 2020;30:6170–7. 32518989 10.1007/s00330-020-06977-5 PMC7280685

[33] Grandmaison G AA Périard D. Systematic screening for venous thromboembolic events in COVID-19 pneumonia. TH Open. 2020;4:e113–5. 32529170 10.1055/s-0040-1713167 PMC7280022

[34] Grillet F. Acute pulmonary embolism associated with covid-19 pneumonia detected with pulmonary CT angiography. Radiol. 2020;296:E186–e188 10.1148/radiol.2020201544 PMC7233384 32324103

[35] Hékimian G. Severe pulmonary embolism in COVID-19 patients: a call for increased awareness. Crit Care. 2020;24:274. 32487231 10.1186/s13054-020-02931-5 PMC7264962

[36] Helms J. High risk of thrombosis in patients with severe SARS-CoV-2 infection: a multicenter prospective cohort study. Intensive Care Med. 2020;46:1089–98. 32367170 10.1007/s00134-020-06062-x PMC7197634

[37] Kerbikov O. High incidence of venous thrombosis in patients with moderate-to-severe COVID-19. Int J Hematol. 2021;113:344–7. 33389655 10.1007/s12185-020-03061-y PMC7778684

[38] Koleilat I. Clinical characteristics of acute lower extremity deep venous thrombosis diagnosed by duplex in patients hospitalized for coronavirus disease 2019. J Vasc Surg Venous Lymphat Disord. 2021;9:36–46. 32593770 10.1016/j.jvsv.2020.06.012 PMC7315975

[39] Le Jeune S. High prevalence of early asymptomatic venous thromboembolism in anticoagulated COVID-19 patients hospitalized in general wards. J Thromb Thrombolysis. 2021;51:637–41. 32812199 10.1007/s11239-020-02246-w PMC7433772

[40] LeBrun DG. Hip fracture outcomes during the COVID-19 pandemic: early results from New York. J Orthop Trauma. 2020;34:403–10. 32482977 10.1097/BOT.0000000000001849 PMC7302077

[41] Llitjos JF. High incidence of venous thromboembolic events in anticoagulated severe COVID-19 patients. J Thromb Haemost. 2020;18:1743–6. 32320517 10.1111/jth.14869 PMC7264774

[42] Longchamp A. Venous thromboembolism in critically Ill patients with COVID-19: Results of a screening study for deep vein thrombosis. Res Pract Thromb Haemost. 2020;4:842–7. 32685893 10.1002/rth2.12376 PMC7272794

[43] Maatman TK. Routine venous thromboembolism prophylaxis may be inadequate in the hypercoagulable state of severe Coronavirus Disease 2019. Crit Care Med. 2020;48:e783–90. 32459672 10.1097/CCM.0000000000004466 PMC7302085

[44] Manjunath MMJ Fraenkel L. Acute pulmonary embolism in critically ill patients with COVID-19. medRxiv. 2020;21:358–64.

[45] Marone EM. Characteristics of venous thromboembolism in COVID-19 patients: a multicenter experience from Northern Italy. Ann Vasc Surg. 2020;68:83–7. 32673648 10.1016/j.avsg.2020.07.007 PMC7358159

[46] Menter T. Postmortem examination of COVID-19 patients reveals diffuse alveolar damage with severe capillary congestion and variegated findings in lungs and other organs suggesting vascular dysfunction. Histopathol. 2020;77:198–209. 10.1111/his.14134 PMC7496150 32364264

[47] Mestre-Gómez B. Incidence of pulmonary embolism in non-critically ill COVID-19 patients. Predicting factors for a challenging diagnosis. J Thromb Thrombolysis. 2021;51:40–6. 32613385 10.1007/s11239-020-02190-9 PMC7327193

[48] Middeldorp S Coppens M. Incidence of venous thromboembolism in hospitalized patients with COVID-19. J Thromb Haemost. 2020;18:1995–2002. 32369666 10.1111/jth.14888 PMC7497052

[49] Mueller-Peltzer K. Pulmonary artery thrombi are co-located with opacifications in SARS-CoV2 induced ARDS. Respir Med. 2020;172:106135. 32947171 10.1016/j.rmed.2020.106135 PMC7483034

[50] Poissy J. Pulmonary embolism in patients with COVID-19: awareness of an increased prevalence. Circulation. 2020;142:184–6. 32330083 10.1161/CIRCULATIONAHA.120.047430

[51] Poyiadji N Cormier P. Acute pulmonary embolism and COVID-19. Radiol. 2020;297:E335–e338. 10.1148/radiol.2020201955 PMC7706099 32407256

[52] Ren B. Extremely high incidence of lower extremity deep venous thrombosis in 48 patients with severe COVID-19 in Wuhan. Circulation. 2020;142:181–3. 32412320 10.1161/CIRCULATIONAHA.120.047407

[53] Soumagne T. Factors associated with pulmonary embolism among coronavirus disease 2019 acute respiratory distress syndrome: a multicenter study among 375 patients. Crit Care Explor. 2020;2:e0166. 32766562 10.1097/CCE.0000000000000166 PMC7339309

[54] Thomas W. Thrombotic complications of patients admitted to intensive care with COVID-19 at a teaching hospital in the United Kingdom. Thromb Res. 2020;191:76–7. 32402996 10.1016/j.thromres.2020.04.028 PMC7182517

[55] Trimaille A. Venous thromboembolism in non-critically ill patients with COVID-19 infection. Thromb Res. 2020;193:166–9. 32707275 10.1016/j.thromres.2020.07.033 PMC7367026

[56] Valle C. Association between pulmonary embolism and COVID-19 severe pneumonia: experience from two centers in the core of the infection Italian peak. Eur J Radiol. 2021;137:109613. 33657476 10.1016/j.ejrad.2021.109613 PMC7903911

[57] van Dam LF. Clinical and computed tomography characteristics of COVID-19 associated acute pulmonary embolism: a different phenotype of thrombotic disease? Thromb Res. 2020;193:86–9. 32531548 10.1016/j.thromres.2020.06.010 PMC7274953

[58] van den Heuvel FMA. Cardiac function in relation to myocardial injury in hospitalised patients with COVID-19. Neth Heart J. 2020;28:410–7. 32643071 10.1007/s12471-020-01458-2 PMC7341471

[59] Ventura-Díaz S. A higher D-dimer threshold for predicting pulmonary embolism in patients with COVID-19: a retrospective study. Emerg Radiol. 2020;27:679–89. 33025219 10.1007/s10140-020-01859-1 PMC7538266

[60] Wang Y. Remdesivir in adults with severe COVID-19: a randomised, double-blind, placebo-controlled, multicentre trial. Lancet. 2020;395:1569–78. 32423584 10.1016/S0140-6736(20)31022-9 PMC7190303

[61] Whyte MB. Pulmonary embolism in hospitalised patients with COVID-19. Thromb Res. 2020;195:95–9. 32682004 10.1016/j.thromres.2020.07.025 PMC7351054

[62] Wichmann D. Autopsy findings and venous thromboembolism in patients with COVID-19: a prospective cohort study. Ann Intern Med. 2020;173:268–77. 32374815 10.7326/M20-2003 PMC7240772

[63] Yu Y. Incidence and risk factors of deep vein thrombosis in hospitalized COVID-19 patients. Clin Appl Thromb Hemost. 2020;26:1076029620953217. 32854513 10.1177/1076029620953217 PMC7457409

[64] Zhang L. Deep vein thrombosis in hospitalized patients with COVID-19 in Wuhan, China: prevalence, risk factors, and outcome. Circulation. 2020;142:114–28. 32421381 10.1161/CIRCULATIONAHA.120.046702

[65] Kollias A. Venous thromboembolism in COVID-19: a systematic review and meta-analysis. Vasc Med. 2021;26:415–25. 33818197 10.1177/1358863X21995566 PMC8024143

[66] Longchamp G. Proximal deep vein thrombosis and pulmonary embolism in COVID-19 patients: a systematic review and meta-analysis. Thromb J. 2021;19:15. 33750409 10.1186/s12959-021-00266-x PMC7942819

[67] Gratz J. Risk of clinically relevant venous thromboembolism in critically ill patients with COVID-19: a systematic review and meta-analysis. Front Med (Lausanne). 2021;8:647917. 33768106 10.3389/fmed.2021.647917 PMC7985162

[68] Huang C. Clinical features of patients infected with 2019 novel coronavirus in Wuhan, China. Lancet. 2020;395:497–506. 31986264 10.1016/S0140-6736(20)30183-5 PMC7159299

[69] Lemos ACB. Therapeutic versus prophylactic anticoagulation for severe COVID-19: a randomized phase II clinical trial (HESACOVID). Thromb Res. 2020;196:359–66. 32977137 10.1016/j.thromres.2020.09.026 PMC7503069

[70] Flumignan RL. Prophylactic anticoagulants for people hospitalised with COVID-19.. Cochrane Database Syst Rev. 2020;10:Cd013739. 33502773 10.1002/14651858.CD013739 PMC8166900

