# Supplementary figures and images for: Risk factors and mortality of pulmonary embolism in COVID-19 patients: Evidence based on fifty observational studies
Source: Medicine (Baltimore). 2022 Nov 11;101(45):e29895. doi: 10.1097/MD.0000000000029895 (PMC9665895; doi:10.1097/MD.0000000000029895)

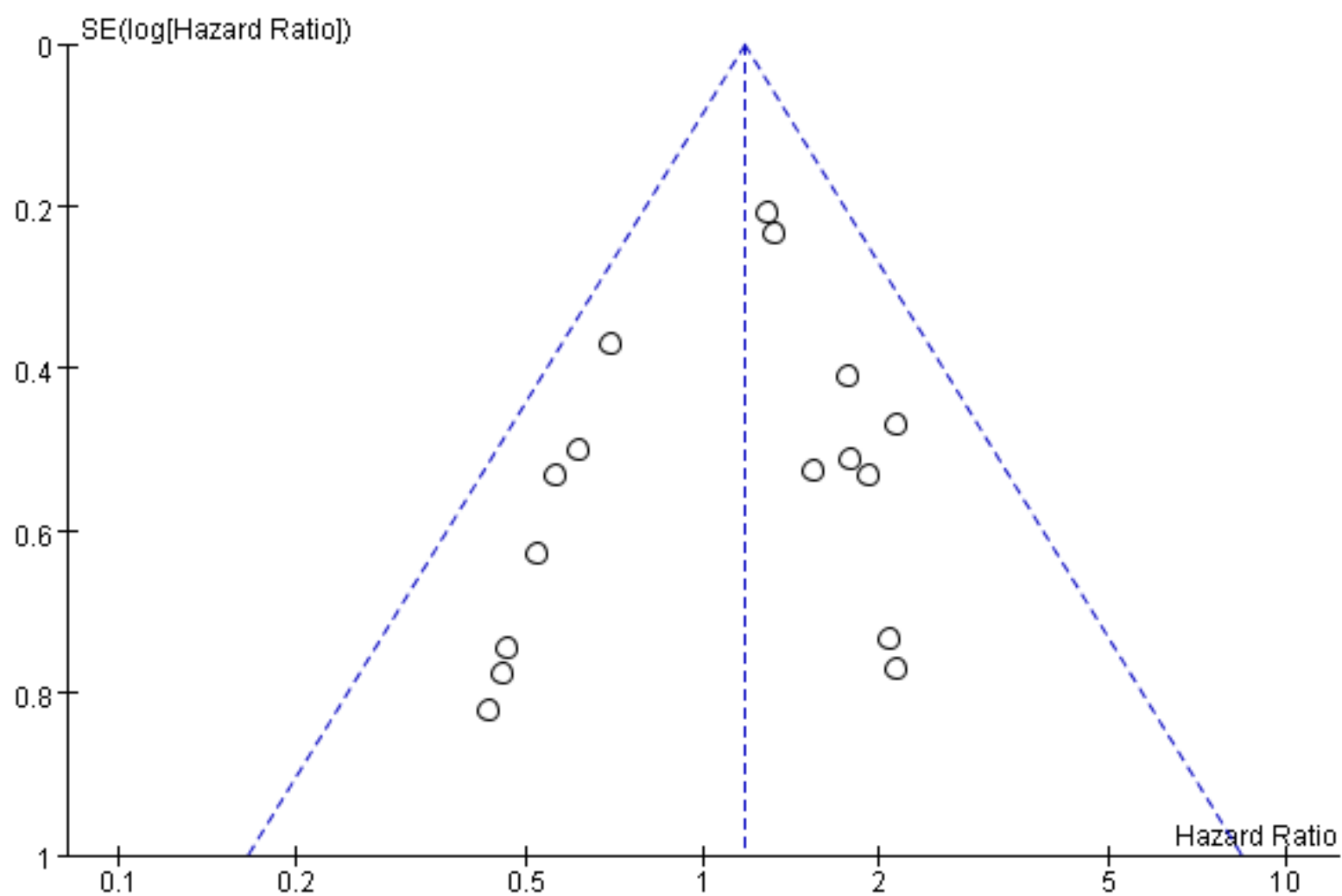

Supplement: Supplementary file 1 [file medi-101-e29895-s001.pdf]
